# Geo-environmental factors and the effectiveness of mulberry leaf extract in managing malaria

**DOI:** 10.1038/s41598-023-41668-3

**Published:** 2023-09-08

**Authors:** Sayantan Pradhan, Samrat Hore, Stabak Roy, Simi Manna, Paulami Dam, Rittick Mondal, Amit Ghati, Trishanjan Biswas, Subhajit Shaw, Supriya Sharma, Waikhom Somraj Singh, Suman Kumar Maji, Sankarsan Roy, Aparajita Basu, Kailash C. Pandey, Soumadri Samanta, Kapil Vashisht, Tuphan Kanti Dolai, Pratip Kumar Kundu, Saptarshi Mitra, Debasish Biswas, Abdul Sadat, Masuma Shokriyan, Amit Bikram Maity, Amit Kumar Mandal, İkbal Agah İnce

**Affiliations:** 1 Department of Sericulture, Raiganj University, North Dinajpur, West Bengal 733134 India; 2 Hematology Department, Nil Ratan Sircar Medical College and Hospital, Kolkata, 700014 India; 3 Department of Statistics, Tripura University, Agartala, Tripura 799022 India; 4 Department of Geography and Disaster Management, Tripura University, Agartala, Tripura 799022 India; 5 Department of Bio-Medical Laboratory Science and Management, Vidyasagar University, Midnapore, West Bengal 721102 India; 6 Department of Microbiology, Barrackpore Rastraguru Surendranath College, Barrackpore, West Bengal 700120 India; 7 ICMR-National Institute of Malaria Research, Sector-8, Dwarka, New Delhi, 110077 India; 8 Department of Pharmacy, Tripura University, Agartala, Tripura 799022 India; 9 District Public Health Centre, Deben Mahata Government Medical College and Hospital, Purulia, West Bengal 723101 India; 10 PH and CD Branch, Office of the Chief Medical Officer of Health, Purulia, West Bengal 723101 India; 11 Department of Microbiology, University of Calcutta, Kolkata, West Bengal 700019 India; 12 Advanced Functional Nanomaterials, Energy and Environment Unit, Institute of Nano Science and Technology (INST), Phase X, SAS Nagar, Mohali, Punjab 160062 India; 13 Department of Microbiology, Santiniketan Medical College, Gobindapur, Muluk, Bolpur, Birbhum, West Bengal 731204 India; 14 Department of Economics, Raiganj University, North Dinajpur, West Bengal 733134 India; 15 Department of Medical Microbiology, School of Medicine, Acibadem Mehmet Ali Aydınlar University, 34752 Ataşehir, Istanbul, Turkey; 16 Department of Otorhinolaryngology, Institute of Post Graduate Medical Education and Research (S.S.K.M. Hospital), Kolkata, West Bengal 700020 India; 17 Centre for Nanotechnology Sciences, Raiganj University, North Dinajpur, West Bengal 733134 India

**Keywords:** Malaria, Microbiology

## Abstract

Malaria prevalence has become medically important and a socioeconomic impediment for the endemic regions, including Purulia, West Bengal. Geo-environmental variables, humidity, altitude, and land use patterns are responsible for malaria. For surveillance of the endemic nature of Purulia’s blocks, statistical and spatiotemporal factors analysis have been done here. Also, a novel approach for the Pf malaria treatment using methanolic leaf extract of *Morus alba* S1 has significantly reduced the parasite load. The EC_50_ value (1.852) of the methanolic extract of *M. alba* S1 with *P. falciparum* 3D7 strain is close to the EC_50_ value (0.998) of the standard drug chloroquine with the same chloroquine-sensitive strain. Further studies with an in-silico model have shown successful interaction between DHFR and the phytochemicals. Both 1-octadecyne and oxirane interacted favourably, which was depicted through GC–MS analysis. The predicted binary logistic regression model will help the policy makers for epidemiological surveillance in malaria-prone areas worldwide when substantial climate variables create a circumstance favourable for malaria. From the in vitro and in silico studies, it can be concluded that the methanolic extract of *M. alba* S1 leaves were proven to have promising antiplasmodial activity. Thus, there is a scope for policy-driven approach for discovering and developing these lead compounds and undermining the rising resistance to the frontline anti-malarial drugs in the world.

## Introduction

Malaria is one of the most fatal vector-borne infectious diseases that affect humans. The disease is clinically and economically troublesome because it is prevalent in poorer countries and regions, significantly hampers socio-economic development. Unicellular protozoan parasites from the genus *Plasmodium* are the causal agents of malaria in humans and other vertebrates. While humans are the primary host for some species of *Plasmodium*, some species can also infect a wide range of vertebrates, including mammals, birds, and reptiles. More than 200 species have been adequately defined of the *Plasmodium* parasite to date, and each species can infect a definite range of hosts^1^. *P. vivax, P. falciparum, P. ovale, P. malariae,* and *P. knowlesi* are the five species of this parasite, which can naturally infect humans to cause malaria in various parts of the world. However, their distribution is not uniform across large areas of the world. The first four species are restricted to humans. Whereas *P. knowlesi* is a species that infects macaques in Southeast Asia but can also cause human malaria and has emerged as a significant public health concern in some areas.

*P. vivax* and *P. falciparum* are the most common species of *Plasmodium* and are responsible for most malaria cases worldwide. *P. vivax* is found mainly in Asia and South America, while *P. falciparum* is found predominantly in sub-Saharan Africa. *P. malariae* and *P. ovale* are less common and found primarily in Africa, although they have also been reported in other regions worldwide.

*P. falciparum* is the deadliest among these five species. Mosquito is generally the insect vector, which causes *Plasmodium* species’ transmission between vertebrate hosts. The vector is the definitive host, not a carrier only, which is the place of *Plasmodium* species’ sexual reproduction. Inside the insect body, the development of a parasite is necessary for the transmission to the following vertebrate host. The *Plasmodium* species’ critical development can be supported by a whole range of insect species depending on individual host-specific parasite species, but anopheline mosquitoes are mainly responsible for the transmission of all five species of *Plasmodium*, which cause malaria in humans. Noticeable genetic flexibility is owned by *Plasmodium* species, which facilitates them to adapt to environmental alterations. This also helps them by giving them the potential to develop resistance fast against therapeutics like antimalarials and to change the specificity of host^1^.

Approximately 247 million malaria cases were there worldwide in 84 malaria-endemic countries in 2021, which was 2 million cases plus from the year earlier including the French Guiana territory. Most of this increase came from the WHO African Region’s countries. Malaria remains endemic in tropical region of Asia and Africa^2,3^. From 2000 to 2019, it was seen that malaria deaths were gradually reduced throughout the world. The reduction is from 897,000 deaths in 2000 to 577,000 in 2015 and 568,000 in 2019. There was an escalation of 10% in malaria deaths in 2020 compared to 2019, which were approximately 625,000 deaths. In 2021, deaths slightly declined to 619,000. At the time of the COVID-19 pandemic situation between 2019 and 2021, disruptions in essential malaria services were responsible for about an extra 63,000 malaria deaths^4–6^. When the inoculation of motile sporozoites takes place into the dermis, the life cycle starts, which travels to the liver afterwards; a hepatocyte is invaded by each sporozoite and multiplies afterwards. The asexual cycle begins when the red blood cells are invaded by thousands of merozoites released into the bloodstream after the liver schizonts burst after 5–21 days depending on parasite species. The beginning of illness is detected consequent to the total asexual parasite load reaching roughly 100 million in circulation.

The development of some parasites occurs in the form of gametocytes, i.e., sexual forms. The formation of gametocytes results within a feeding anopheline mosquito due to the form of an ookinete and an oocyst after that through sexual development after reproduction inside the gut of the mosquito. Sporozoites are liberated after the oocyst bursts, which the salivary glands receive for the expected inoculation at the next blood feed. More or less, 1 month is needed for the entire cycle. In an adult round, about 2% of parasitemia corresponds to 10^12^ parasite burden in a total body^7^.

Environmental factors affect the transmission intensity, seasonality, and geographical distribution of malaria, and together with the vector, the human and the parasite compose the eco-system of malaria^8^. Meteorological factors, i.e., temperature, humidity and precipitation, are the primary environmental determinants of malaria. Temperature impacts vector and parasite development and thus is a significant constraint on the geographical suitability to malaria^9,10^. The overall correlation between mosquito density and rainfall has been frequently illustrated; rainfall offers mosquito breeding locations and raises the humidity, which improves their survival^11^. The difference in climatological conditions, mostly temperature, humidity and rainfall, regulates the spatiotemporal configurations of malaria through their effects on both the *Plasmodium* parasite and the *Anopheles* vector^12^. Thus, malaria control strategies must focus on the ecological roles, especially in areas where this life-threatening disease is a major public health concern^13^.

Drugs named primaquine and chloroquine are used for the treatment of uncomplicated *P. vivax* malaria. For 14 days, primaquine is taken to resist the relapse caused by Pv malaria, while chloroquine is taken for 3 days. Primaquine cannot be given to children under 1-year, pregnant mothers and patients who have G-6PD deficiency. To treat patients with uncomplicated Pf infections, artemisinin-based combination therapies (ACTs), such as, artemether-lumefantrine, artesunate-amodiaquine, artesunate-sulfadoxinepyrimethamine (SP) are used for 3 days^5^. The treatment regimen for Pf malaria involves administering a single dose of primaquine on the 2nd day to clear the gametocyte as per the patient’s body weight. If patients suffering from mixed infections of both Pv and Pf malaria, ACT is used for 3 days, and a daily dose of primaquine is prescribed for 14 days as per the patient’s body weight^14^.

The emergence of resistance to antimalarial drugs is comparatively a recent phenomenon, although there is a long history of the use of antimalarial drugs. In Thailand, there was the first appearance of the chloroquine-resistant forms of *P. falciparum* malaria in 1957^15^. Artemisinin-resistant malaria has become a pressing issue worldwide in recent years. The first reports of resistance to artemisinin-based combination therapy (ACT) came from western Cambodia and the Thailand-Cambodia border in 2002–2004. Since then, the problem has spread to other regions, and there is growing concern about the potential impact on global malaria control efforts^16^*.* The rise of artemisinin resistance in Southeast Asian countries has led to an increased treatment failure rate, as the effectiveness of both artemisinin and its partner drugs has diminished^17^. This resistance is particularly pronounced in the Greater Mekong sub-region, where it has spread widely and intensified by resistance to the partner drug, leading to the collapse of artemisinin combination therapies. This development is concerning as it undermines efforts to control and eliminate malaria in the affected regions^18^. So, there is a safe, effective, and affordable triple ACTs (TACTs) alternative approach formed by artemisinin and two of the existing partner drugs for tackling this global issue of resistance to ACT.

To construct a binary logistic regression model that uses four climatic variables—relative relief, temperature, humidity, and rainfall—to predict the endemic status of blocks in the Purulia district, were considered as response variables. In the model, endemic blocks were coded as ‘1’ and non-endemic blocks as ‘0’. By leveraging these variables, the model can help to identify areas at risk of endemic malaria and provide guidance for public health officials and policymakers seeking to prevent and control the spread of the disease. A model framework that utilises four explanatory variables can help to predict the level of endemic malaria in a given block without the need for door-to-door data collection. This predictive model can help local authorities to take proactive measures to reduce malaria cases before significant climatic variables create a favourable environment for malaria transmission. By leveraging these predictive insights, administrators can take targeted actions to prevent the spread of malaria in vulnerable areas, ultimately helping to improve public health outcomes. The emergence of resistant strains posing an additional challenge to malaria control. Thus, new drugs should be introduced to combat such resistant strains without having adverse side effects. The Indian holy literature Rigveda and Atharvaveda, two of the four Vedas (Hindu holy scripture), outline the history of plants and its uses^19^. Additionally, ethanobotanist revealed that plants have hiostorically been used to fight malaria^20^. Quinine and artemisinin, the two major antimalarial drugs, are both screened from bark of *Peruvian Cinchona* and Chinese antipyretic *Artemisia annua* respectively^21^. Thus, without any doubt the plants represent a vast bioresource of novel anti-malarial drugs. In malaria-endemic areas, especially in Africa, many people rely on herbal medicines as the first line of treatment due to its low-cost, availability, perceived effectiveness, low side effect, faith in traditional medicines and its accesibility^22^. In an effort to screen and identify the lead anti-malarial compound we have investigated the methanolic leaf extract of *Morrus alba* S1 and determine its efficacy against 3D7 strain of Pf malaria through in vitro*, *in silico analysis that might be helpful for public health by playing a potentially effective role in Pf malaria treatment^23–27^.

## Methods

### Spatiotemporal factors

The four key climatic variables—namely relative relief, temperature, humidity, and rainfall, which—are widely recognised as critical determinants of malaria incidence^13,28–34^. The relative relief of the Purulia district was obtained from the Radiometric Terrain Correction Alos Palsar DEM with a resolution of 12.5 m. The DEM data were collected from the NASA Earth Data server. Fishnet tool of ArcMap calculated the relative relief of the Purulia District. The temperature and rainfall data used in this study were collected from the World Bio-Climatic Data Portal which is using remote sensing technology". NASA’s Climate Data Services (CDS) were the source of Humidity data for the study area.

The spatial resolution, pixel depth, and projection system (UTM) of all four geo-climatic variables (relative relief, temperature, humidity, and rainfall) were verified using data from NASA’s Climate Data Services (CDS) and the World Bio-Climatic data portal. The humidity data for the study area was obtained from NASA's CDS with a spatial resolution of 1 km × 1 km. A spatial zonation map was generated using the Inverse Distance Weightage (IDW) method in ArcGIS v.10.8 and Global Mapper v.22 software, based on spatially rectified images of the geo-climatic variables^35^.

### Statistical analysis

The study conducted a correlation analysis among four spatiotemporal factors: elevation, temperature, rainfall, and humidity, to identify significant correlations at 1% and 5% levels of significance. Based on the endemicity of each block in the Purulia district, a binary logistic regression model was structured with the aforementioned environmental variables as explanatory variables. This model framework can predict the endemicity of a block beforehand, provided the respective environmental variables are known.

### Preparation of leaf extract

The collection of plant material complied with relevant institutional, national, and international guidelines and legislation. Such plant samples were collected in the close coordination with Phulpahari Sericulture Complex, Midnapore. The plants voucher specimen was deposited in a publicly available central national herbarium of the Botanical Survey of India (Specimen No: P/M/PM/SP-01). Living material is growing at Phulpahari Sericulture Complex nursery for future research. The plant material was prepared from fresh and healthy collected leaves of *Morus alba* S1 from Phulpahari Sericulture Complex, Midnapore. The freshly collected leaves were subjected to thorough wash under running tap water followed by distilled water before they were cut into small bits. Then the leaves were dried under sunlight. After that, the dried leaves were finely powdered using a mixture grinder and preserved in an airtight container. The dried powdered leaf sample of 20 g was dissolved in 200 ml of methanol in amber colored bottle and stirred for 8 h in magnetic stirrer. Then the sample was filtered using Whatman filter paper 1. The filtrate sample was concentrated with rotary evaporator at 65 °C under reduced pressure. The concentrated sample was further dried in hot plate at 40 °C for overnight. The dried sample was further kept in -20 °C for further use^36,37^.

### GC–MS analysis

The GC–MS analysis of the methanol leaf extract was performed using GC-MSQP10 Ultra, equipped with a fused silica column, packed with Rxi-1 ms capillary column. 99.99% helium gas was used as the carrier gas. The injector temperature was 310 °C. The column oven temperature was set to 80 °C for 2 min and increased to 150 °C, 200 °C and 260.0 °C subsequently each for 4 min and the final temperature rose to 310 °C for 18 min. The injection mode was split, and the total flow was 10 ml/min where the column flow was 0.64 ml/minute. The purge flow was 3 ml/ min with split ratio of 10.0. The spectral detection gained at 1.03 kV + 0.00 kV of ionisation energy. The acquisition mode of the sample is scan with 0.30 scan time and 3333 scan speed; the fragments range from 50 to 800 m/z. The test sample constituents were analysed by considering their retention time (minute), peak height, peak area and mass spectral patterns with the spectral patterns present in the standard database.

### In vitro antimalarial activity

*P. falciparum* strain 3D7 [sensitive to the standard drug chloroquine (CQ)] and RKL9 (resistant to CQ) were cultured in RPMI media supplemented with AB^+ve^ human serum (10%) and A^+ve^ erythrocyte at 5% hematocrit. Synchronisation of the parasite was done at the early trophozoite stage with 5% sorbitol. Briefly, a 1:9 ratio of pellet and sorbitol was kept at room temperature for 5 min^38^. The supernatant was removed by centrifugation, and the pellet was washed thrice in complete media (RPMI-1640 supplemented with 10% AB^+ve^ human serum). To this synchronised culture, complete media was added to make a 1:9 ratio of infected RBC (5% parasitaemia and 10% hematocrit) and media, and the experiment was planned. The plant extract samples were dissolved in DMSO to obtain a stock solution of 1 mg/ml. The stock solutions were further diluted with RPMI-1640 to get the different concentrations of 50–0.8 μg/ml in serial dilution and dispensed in the 96-well flat-bottomed microplates. The experiment was set up with a modified WHO MARKIII protocol^39^. Briefly, parasite cultures were treated with different concentrations of samples, and incubated at 37 °C in a gas mixture of 90% nitrogen (N), 5% carbon dioxide (CO2) and 5% oxygen (O_2_) for 24 h, and incubation continued till the schizont growth was 10% in control. The blood smear was prepared from all the wells and stained with Giemsa stain. Schizonts with three or more merozoites were counted. The result was analysed using dose–response curves by non-linear regression analysis performed with HN-NonL in Regression Analysis^40^.

### In silico study

Studies for molecular docking were performed using Biovia Discovery Studio 2021 (v21.1.0.20298) software to determine the possible interaction modes of the target compounds in the dihydrofolate reductase (DHFR). The X-ray crystallographic structure of the enzymes DHFR was downloaded from the protein data bank (https://www.rcsb.org/structure/2BL9), PDB codes: 2BL9 respectively. While the bound substances (ligand and cofactors) and solvent molecule associated with the receptor were removed from the target protein (DHFR; 2BL9) to vacate the binding site for the interaction. The chemical structure of the molecules was drawn with ChemDraw ultra-Version 8.0 and further pre-optimized for docking. Furthermore, the prepared ligands were docked in silico with the DHFR inhibitors using Biovia Discovery Studio 2021 (v 21.1.0.20298)^41–45^.

### Ethics declaration

This study was allowed by Zilla Swasthya Bhawan, Purulia, Govt. of West Bengal (Memo No. 2041; Dt. 03.11.2020). The ethical approval (No. RUHECRP001) was also acquired from the Raiganj University Human Ethical Committee (RUHEC). The data were anonymized and combined at the Zilla Swasthya Bhawan without any individual patient information. All protocols were executed as per the pertinent guidelines and regulations. Informed consent was collected individually from all the participants or if the participants are < 18, from a parent and/or legal guardian.

## Results

### Geo-environmental externalities

**Figure 1:**
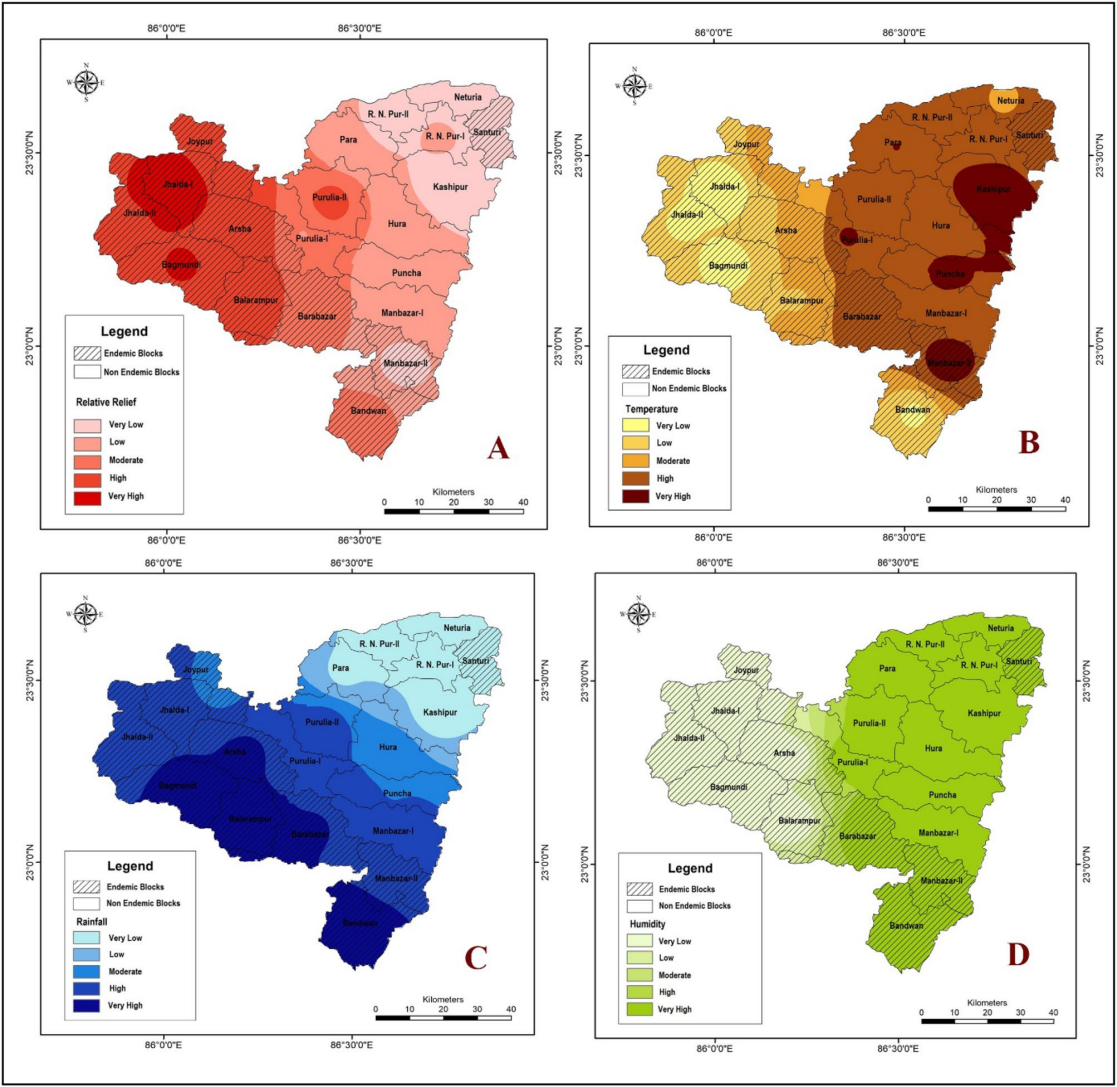
Spatial zonation of spatiotemporal variables (**A** elevation, **B** temperature, **C** rainfall and **D** humidity (Source: generated using ArcMap v.10.8, 2022).

Spatial distribution of the Purulia district according to the average measure of four different spatiotemporal factors, i.e. elevation, temperature, rainfall and humidity, were established and classified into five different zones very low, low, moderate, high and very high. Empirical evidence indicates that areas with elevations classified as very high (600 m) to high (400 m) have a higher incidence of malaria, as demonstrated in the maximum malaria endemic blocks of Jhalda-1, Bagmundi, Jhalda-II, Joypur, Arsha, and Balarampur. (Fig. 1A). The result depicts that higher elevation of Purulia district has an associations with endemic nature of these community development blocks. According to the Indian Meteorological Department of India (2021), the average annual temperature of the Purulia district varies from 21 to 38 °C. *P. falciparum* cannot complete its growth cycle in the *Anopheles* mosquito and thus cannot be transmitted at temperatures below 20 °C (68°F). Lunde et al. showed that the optimal temperature for malaria transmission is below 30 °C^46^. Based on observation, there appears to be a correlation between the average temperature and the incidence of malaria cases in certain regions. Specifically, in the Jhalda-I, Jhalda-II, Bagmundi, Bandwan, Arsha, Balarampur, and Joypur blocks, the average temperature has been found to range from very low (25 °C) to low (30 °C), which appears to be synchronized with a higher incidence of malaria cases (Fig. 1B).

Purulia district is situated in a tropical savanna climate (Aw) and experiences significant rainfall during the monsoon season—the average rainfall of the district is from 1100 to 1500 mm, which influences the growth of malaria cases. Our study revealed that Arsha, Balarampur, Bagmundi, Barabazar, and Bandwan blocks received the highest rainfall, and remarkably, they fall under the endemic category (as shown in Fig. 1C). *Anopheles* mosquitoes thrive in low humidity conditions^47^. Our analysis indicated that Arsha, Bagmudi, Balarampur, Jhalda-I, Jhalda-II, and Joypur blocks have lower humidity levels, which may help *Anopheles* mosquitoes to survive and transmit the malaria parasite (as shown in Fig. 1D).

The correlation test between all four spatiotemporal variables is carried out and reported in the following table (Supplementary Table S1). It has been observed that all four spatiotemporal variables significantly correlated with each other.

To predict the endemic nature of a block, we have carried out binary logistic regression over any one of the above four significant spatiotemporal variables while coding 10 endemic blocks as ‘1’ and the remaining 10 non-endemic blocks as ‘0’ (Table 1). As the above are significantly correlated, we can consider any of the four as an explanatory variable. In this study, we have considered temperature as the explanatory variable because of easy to measure compared to other variables. The predicted binary logistic regression model and respective fitted model table are as follows:

1 $$P(Y = 1) = \frac{1}{{1 + e^{ - (262.2979 - 10.0701*temperature)} }}$$

**Table 1:** Binary logistic regression fitted table.

Variable	Co-efficient	Standard Error	*p*-value
Constant	262.2979	121.9345	0.0315*
Temperature	− 10.0701	4.6759	0.0313*

N.B. *Correlation is significant at the 0.05 level (Source: Prepared by the authors, 2022 SPSS v.24).

The above model (Eq. 1) will help to find out the endemic nature of a particular block of the Purulia district in future when we can easily observe the average monthly temperature. Before substantial climate variables create a circumstance where malaria is likely to occur, this predicted model will assist the administration in reducing the number of malaria-affected cases.

### GC–MS analysis

**Figure 2:**
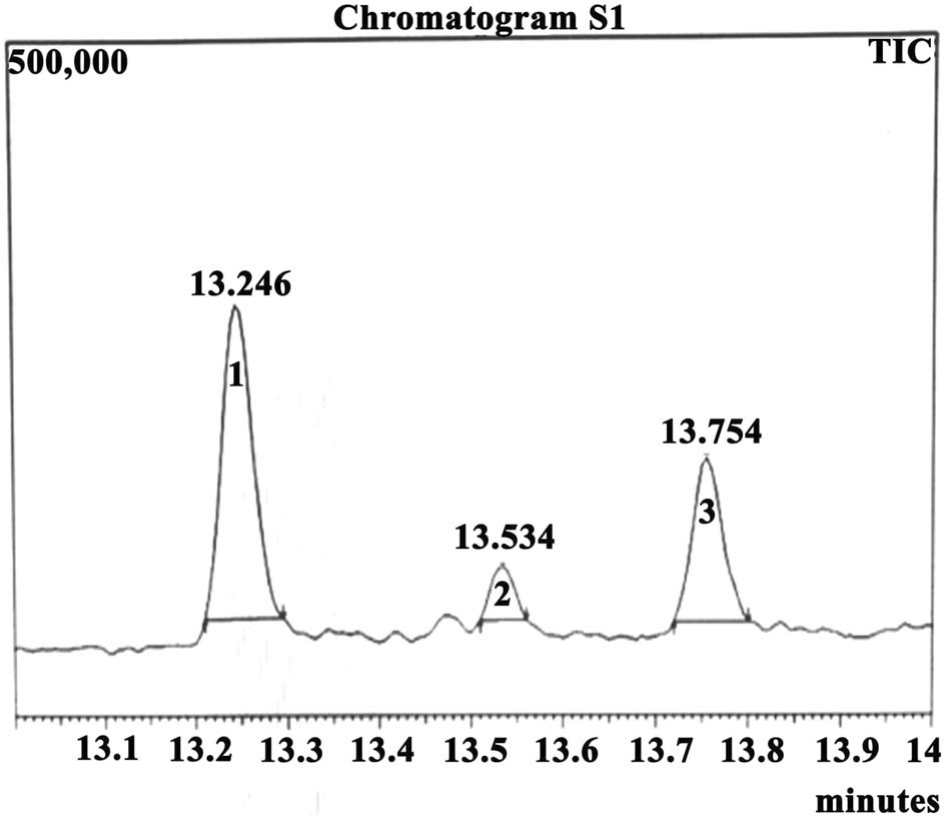
GC–MS chromatogram of the methanolic leaf extract of *Morus alba* S1.

GC–MS is the combined, reliable and accurate analytical technique to determine and identify the various compounds present in leaf extract. GC–MS analysis of the methanolic leaf extract of *M. alba* S1 detected three different phyto-chemicals. The chromatogram of GC–MS showed three visible peaks which were recognised by their corresponding peak, retention time, peak area (%), height (%) and with mass spectral fragmentation patterns as specified through the known compounds described by the library of the National Institute of Standards and Technology (NIST) (Fig. 2). The three compounds detected in GC–MS were identified as 1-Octadecyne (C_18_H_34_); 6-Octen-1-ol, 3,7-dimethyl-, propanoate (C_13_H_24_O); and Oxirane, tetradecyl-SSHexadecane,1,2-epox (C_16_H_32_O) which corresponds with the NIST Library search (Table 2). The structure of these three compounds was analysed and revealed in Supplementary Fig. S1. The details of the three phyto-compounds of the methanolic extracts from leaves of *M. alba* S1 are presented in Table 2 along with their retention time. Out of these three compounds, two compounds showed the highest percentage (%) of area, i.e.,1-Octadecyne (61.40%) and Oxirane, tetradecyl SSH exadecane,1,2 epox (30.67%) and were chosen for further in vitro antiplasmodial studies.

**Table 2:** Phyto-chemistry of the methanolic leaf extract of *Morrus alba* S1 as revealed through GC–MS analysis.

Peak No	Compound Name	Molecular formula	Molecular weight	Retention time (minute)	Area	Area (in %)	Height	Height (in %)
1	Oxirane, tetradecyl-	C_16_H_32_O	240.4247	13.754	263,372	30.67	118,614	30.4
2	6-Octen-1-ol, 3,7-dimethyl-, propanoate	C_13_H_24_O	212.3285	13.534	68,042	7.92	39,066	10.12
3	1-Octadecyne	C_18_H_34_	250.4626	13.246	527,189	61.40	228,182	59.14

### In vitro study

To provide an initial characterization of the in vitro antiplasmodial activity of the leaf extract was checked on *P. falciparum* strain 3D7 and RKL9 and the results were presented in Table 3. In this in vitro study, the half maximal effective concentration (EC_50_) value of CQ with 3D7 strain (CQ sensitive) was 0.998, EC_50_ value of CQ with RKL9 strain (CQ resistant) was 11.078 and the EC_50_ value of our test drug, i.e., *M. alba* S1 methanolic leaf extract was 1.852. Again, EC_99_ value of CQ with 3D7 strain (CQ sensitive) was 11.915, EC_99_ value of CQ with RKL9 strain (CQ resistant) was 110.723 and EC_99_ value of the test drug *M. alba* S1 methanolic leaf extract was 47.880. The R^2^ value of CQ with 3D7 strain (CQ sensitive) was 0.9115, CQ with RKL9 strain (CQ resistant) was 0.9591 and the test drug was 0.9681.

**Table 3:** *In-vitro* antimalarial activity of plant extracts against CQS (3D7) strain of *P. falciparum*.

Sample Id	Polynome	EC50	EC90	EC95	EC99	R^2^
S1	3	1.852	23.029	37.935	47.880	0.9681
S1-D	3	1.928	29.658	39.935	47.099	0.9453
CQ with 3D7 (CQ sensitive)	3	0.998	1.849	10.883	11.915	0.9115
CQ with RKL9 (CQ resistant)	3	11.078	7.714	9.865	110.723	0.9591

### In silico study

Study of molecular docking of the target compounds, which was carried out against 2BL9 enzyme and is displayed in Table 4. Compounds 1-octadecyne **(1)**, tetra decyl oxirane (**2**) and gallic acid (**3**) with the best binding were visualised and analysed using Discovery Studio. The binding affinity values for all the target compounds range from − 6.031 to − 8.029 kcal/mol with the target site against the DHFR enzyme. The target compounds **1** (− 8.029 kcal/mol), **2** (− 7.997 kcal/mol) and **3** (− 6.031 kcal/mol) showed remarkable binding affinity, which was more significant than the binding affinity of recommended drugs; chloroquine **(std)** (− 7.813 kcal/mo) while gallic acid shows lesser binding affinity than the other two tested compounds and recommended drug.

**Table 4:** Molecular docking study of the target compounds with DHFR.

Compound codes	Binding Affinity (Kcal/mol)	Hydrogen bond		Hydrophobic bond Amino acid	Neutral amino acid
		Amino acid	Bond length (A˚)		
**1**	− 8.029	**–**	**–**	TYR A: 167, LEU	
				A:162, ILEA:10,	
				PHE A:171, ALA	NDP A:
				A:12, TYR A:179,	1239,
				ILE A:13, ILE A:	CP A:61,240
				172, LEU A:45,	
				ALA A:15, TRP	
				A:47, ALA A:12	
**2**	− 7.997	**–**	**–**	ALA A:15, CYS A:	
				14, TYR A: 179,	
				ALA A:12, ILE A:	NDP A:
				172, VAL A: 112,	1239, CP A:
				LEU A: 158, ILE	61,240
				A:10, LEU A: 162,	
				CYS A: 170, VAL	
				A:110	
**3**	− 6.031	CP A: 6124	2.30	ILE A:172, ALA A:12, ILE A:13	**–**
		TYR A: 11 TYR A:179	3.10 2.75		
		TYR A: 11	2.85		
**Std**	− 7.813	ILE A:13	2.93	ILE172, ILE188,	**–**
				ILE A:10, CYS	
				A:182, ALA A: 12,	
				VAL A: 178, TYR A	
				:179, TYR A: 56	

N.B. “**–**” indicates no interactions.

**Figure 3:**
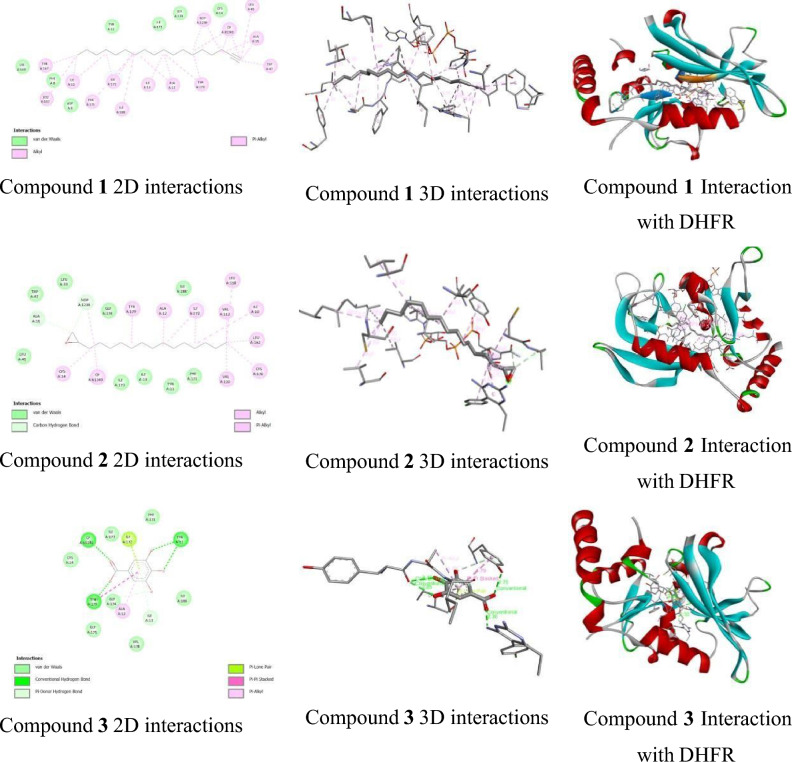
3D and 2D Interactions between synthesized compounds 1, 2 and 3 with the target site of the p38 MAP kinase enzyme.

Compound **1** (Fig. 3) accounts for no hydrogen bond with the target site. While it formed a hydrophobic bond with TYR A: 167, LEU A:162, ILEA:10, PHE A:171, ALA A:12, TYR A:179, ILE A:13, ILE A: 172, LEU A:45, ALA A:15, TRP A:47, and ALA A:12 of the target site. Compound **1** formed neutral interaction with the target site CP A:61,240, and NDP A:1239. Compound **2** (Fig. 3) formed no hydrogen bond with the target site while it interacts with ALA A:15, CYS A: 14, TYR A: 179, ALA A:12, ILE A: 172, VAL A: 112, LEU A: 158, ILE A:10, LEU A: 162, CYS A: 170, and VAL A:110 to formed a hydrophobic bond with the target site. Similarly, tetra decyl oxirane formed neutral interaction with the target site CP A:61,240, and NDP A:1239. Compound **3** (Fig. 3) formed four hydrogen bonds with the target site. The –C=O group and OH of the compound acted as a hydrogen bond acceptor and formed four hydrogen bonds with CP A: 6124, TYR A: 11, TYR A:179, and TYR A: 11 of the targets. Compound **3** formed hydrophobic bonds with ILE A:172, ALA A:12, and ILE A:13 of the target sites.

**Figure 4:**
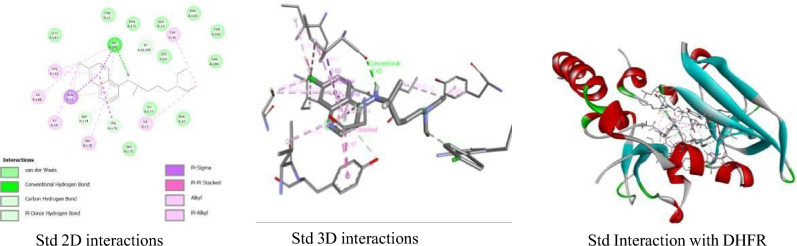
3D and 2D Interactions between chloroquine (std) with the target site of the DHFR enzyme.

The recommended drugs, chloroquine (std) (Fig. 4), account for one hydrogen bond (**ILE A:13**) while hydrophobic bonds were observed with ILE172, ILE188, ILE A:10, CYS A:182, ALA A: 12, VAL A: 178, TYR A :179, and TYR A: 56 and account no hydrophilic interaction with the target site of DHFR.

## Discussion

**Figure 5:**
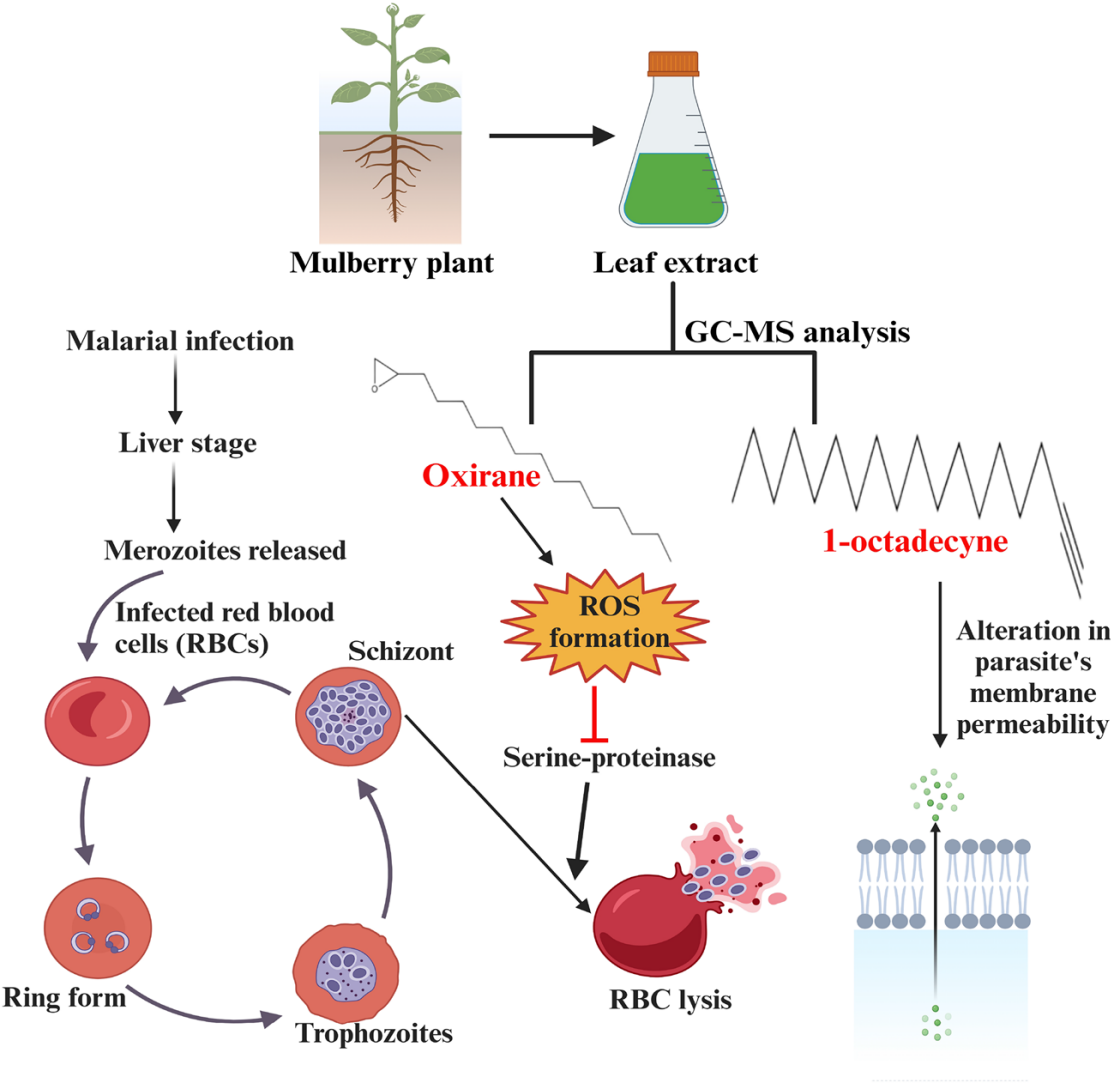
Mode of action of anti-malarial compounds screened from *Morus alba* S1 leaf extract.

The Purulia district has been classified into five different zones from very low to very high in malaria-endemic based on various spatiotemporal factors. Maximum malaria-endemic areas are in extremely high to high elevations, including Jhalda-1, Bagmundi, Jhalda-II, Joypur, Arsha, and Balarampur. Although below 30 °C is the ideal temperature for malaria transmission, The average temperature varies between very low and low in the Jhalda-I, Jhalda-II, Bagmundi, Bandwan, and Arsha, Balarampur, Joypur blocks, which coincides with a high number of malaria case. Those blocks are also identified as high rainfall and low humidity zone, making malaria-prone endemic blocks. According to such endemic and non-endemic nature, the binary logistic regression model has been framed by considering temperature as a significant spatiotemporal factor (Supplementary Table S1). From the literary sources, it has been consistently observed that spatiotemporal factors play a critical role in the increased prevalence of malaria^13^. The predicted model will help the administration in minimizing the number of cases of malaria before significant climatic variables lead to a situation where malaria is likely to arise. 1-octadecyne and oxirane are the two compounds, as revealed from the GC–MS study, that might be responsible for the antimalarial therapeutic ability of the methanolic extract of *M. alba* S1 leaves. The GC–MS analysis of the methanolic extract of *M. alba* S1 leaves has revealed two major peaks with retention time of 13.246 (min) and at 13.754 (min). The GC–MS reference catalogue analysis identified the compounds as 1-Octadecyne (peak 1 with molecular weights of 250) and Oxirane (peak 3 with molecular weights of 240). The detailed result of GC–MS analysis was shown in Fig. 2, Supplementary Fig. S1 and Table 2. The earlier studies found that these compounds of other origins exhibited antimalarial and antioxidant activities^48,49^. 1-octadecene was reported to have therapeutic applications representing potent antimalarial, antioxidants and antibacterial activities^49,50^. It was assumed that 1-octadecene may cause alteration in the cell membrane permeability in parasites resulting in leakage of metabolites and ions^51^. The synthetic derivatives of oxirane were found to be promising antiplasmodial compound which mechanistically might inhibit serine protease of *P. falciparum* via generation ROS^48^ (Fig. 5). Therefore, the leaf of *M. alba* contained both of these antimalarial compounds together, which could be utilised in potential therapeutic applications in antimalarial drug developments^26^.

As a standard in the in vitro study to co-relate the efficacy of the said methanolic extract of *M. alba* S1 leaves against malaria, the CQ-sensitive strain 3D7 and the CQ-resistant strain RKL9 were used. Here CQ was used as a standard drug. The result was obtained from the experiment, which was designed with the modified WHO MARKIII protocol in the 96-well flat-bottomed microplates (Supplementary Fig. S2A)^39^. From the Giemsa-stained blood smears (Supplementary Fig. S2B) of all the wells, these results were evaluated and analysed through dose–response curves by non-linear regression analysis using HN-NonLin Reg. Analysis^40^. Here, half maximal effective concentration of the (EC50) value of CQ with 3D7 strain (CQ sensitive) was 0.998 and 1.852 was the EC50 value of our test drug, i.e., *M. alba* S1 methanolic extract from leaves, which was quite close to CQ. Again, EC99 value of CQ with RKL9 strain (CQ resistant) was 110.723 and 47.880 was the EC99 value of the test drug that is more effective than the EC99 value of CQ with RKL9 strain. From the R^2^ value of the methanolic leaf extract, which was 0.9681, it can be suggested that the said methanolic leaf extract of *M. alba* S1 leaves has a significant effect against Pf malaria (Table 3). To sum it up, the result shown by the methanolic extract of *M. alba* S1 leaves is efficient against the 3D7 strain of Pf malaria parasite^23,24^. The activity can be improved by creating better extraction conditions. Also, microscopically, parasite clearance was found after treatment with the methanolic leaf extract, which demonstrates the methanolic leaf extract’s potential efficacy against malaria (Supplementary Fig. S2C). The earlier studies reported the medicinal benefits of mulberry fruit and syrup in the treatment of urine inconsistency, constipation, dizziness, throat infection, tinnitus, dyspepsia, melancholia, fever, depression and endemic malaria^52^. In a separate study, it was reported that methanolic extract of *M. alba* L. showed antimalarial activity against *P. falciparum* 3D7^53^. It was also reported that, alkaloids isolated from the *Morus* plant possess antimalarial properties^54^.

In one of our earlier studies, we found the gallic acid to be an essential compound in mulberry leaf extract, which extract was analysed for the antibacterial activity^55^. It was reported previously that gallic acid has adequate antimalarial activity^56^. Based on molecular docking studies, gallic acid has showed remarkable binding affinities through hydrophobic bonds with the target sites of DHFR (Table 4). Here, our two target compounds, i.e., 1-Octadecyne and Tetra decyl oxirane, had shown remarkable binding affinities which were greater than the binding affinity of recommended drugs, such as chloroquine (Table 4 and Figs. 3 and 4). The presence of a similar residual amino acid (ILE A:172 & ALA A:12) in the three targeted compounds against the target sites of DHFR is shown in Table 4 highlighted in blue color. While ILE A:13 residue shows the involvement in target compounds 1 and 3 against the target site of DHFR highlighted green color (Table 4). The higher binding affinity value and the involvement of residual amino acids in the targeted compounds can act as potent anti-malarial agents in the DHFR pathway. This indicates that the said methanolic extract of *M. alba* S1 leaves has significant antimalarial activity. Antifolate is one of the widely used drugs to control malaria due to the dihydrofolate reductase enzyme which is required for the reduction of dihydrofolate to tetrahydrofolate. Inhibition of folic acid synthesis is one of the major actions of antimalarial agents; this played a key role in the amino acid and nucleotide synthesis. The nuclear division of *Plasmodium* species was inhibited by the antifolate agents thus at the schizont stages of the erythrocyte and hepatocytes. A significant target for antimalarial agent development^56,57^.

Our study has some limitations. The present report uses routine data that are only representative of the populations that seek care and are laboratory tested. It therefore excluded variations in testing frequencies, and infections that are asymptomatic. Geographical heterogeneity could be another limitation of our study, as the frequency of asymptomatic spread may varies as per the epidemiological situation, transmission rates, and subsequent host immunity. Thus, further work may look into leveraging information from both sources to better understand the relationship between the data sources and how well they reflect the different components of the transmission system. Establishing these relationships in cooperation with researchers, politicians, and healthcare practitioners would also be important to better develop the thresholds used for defining risk categories.

## Conclusion

The prevalence of malaria in Purulia district, West Bengal, India, is influenced by various geo-environmental factors such as temperature, humidity, rainfall, vegetation, and topography. Anthropogenic activities such as deforestation, urbanisation, and agricultural practices also play a significant role in spreading the disease. Effective measures to control the incidence of malaria in this region would require a comprehensive understanding of the interactions between these factors and their impact on the disease. All these are tried to analyse in this study which will help the policymakers to strengthen the epidemiological surveillance of malaria. This study also demonstrates the chemico-biological efficacy of Morus alba S1 leaf extract against the 3D7 and RKL9 strains of *P. falciparum* malaria through both in-vitro and in-silico analysis, as revealed by a positive interaction between participating target components of the *M. alba* S1 leaf extract. This was also proved through GC–MS analysis and molecular docking studies, as the common interacting target components were found to be same in both studies. It demonstrates that Mulberry leaf extract is potentially effective against Pf malaria. In conclusion, further examinations using bioassay-guided isolation of the active compounds along with their clinical trials are needed for drug development and to establish such lead compounds as potential antimalarial drugs.

### Supplementary Information

Supplementary Information.

## Data Availability

A complete de-identified patient dataset will be available to the researcher upon request. Individuals wishing to access the data should send a request to the tkdolai@hotmail.com or amitmandal08@gmail.com, or ikbal.agah.ince@gmail.com.
