# Supplementary material for: Geo-environmental factors and the effectiveness of mulberry leaf extract in managing malaria
Source: Sci Rep. 2023 Sep 8;13:14808. doi: 10.1038/s41598-023-41668-3 (PMC10491663; doi:10.1038/s41598-023-41668-3)
Supplement: Supplementary file 1 — Supplementary Information. [file 41598_2023_41668_MOESM1_ESM.doc]

# Geo-Environmental Factors and the Effectiveness of Mulberry Leaf Extract in Managing Malaria

*Sayantan Pradhan^1,2,#^, Samrat Hore^3,#^, Stabak Roy^4^, Simi Manna^5^, Paulami Dam^1^, Rittick Mondal^1^, Amit Ghati^6^, Trishanjan Biswas^1^, Subhajit Shaw^1^, Supriya Sharma^7^, Waikhom Somraj Singh^8^, Suman Kumar Maji^9^, Sankarsan Roy^10^, Aparajita Basu^11^, Kailash C. Pandey^7^, Soumadri Samanta^12^, Kapil Vashisht^7^, Tuphan Kanti Dolai^2^, Pratip Kumar Kundu^13^, Saptarshi Mitra^4^, Debasish Biswas^14^, Abdul Sadat^1^, Masuma Shokriyan^15^, Amit Bikram Maity^16,*^, Amit Kumar Mandal^1,17,*^, & İkbal Agah İnce^15,*^*

^1^Department of Sericulture, Raiganj University, North Dinajpur, West Bengal 733134, India. ^2^Hematology Department, Nil Ratan Sircar Medical College & Hospital, Kolkata 700014, India. ^3^Department of Statistics, Tripura University, Agartala, Tripura 799022, India. ^4^Department of Geography and Disaster Management, Tripura University, Agartala, Tripura 799022, India. ^5^Department of Bio-Medical Laboratory Science & Management, Vidyasagar University, Midnapore, West Bengal 721102, India. ^6^Department of Microbiology, Barrackpore Rastraguru Surendranath College, Barrackpore, West Bengal-700120, India. ^7^ICMR-National Institute of Malaria Research, Sector-8, Dwarka, New Delhi 110077, India. ^8^Department of Pharmacy, Tripura University, Agartala, Tripura 799022, India. ^9^District Public Health Centre, Deben Mahata Government Medical College and Hospital, Purulia, West Bengal 723101, India. ^10^PH & CD Branch, Office of the Chief Medical Officer of Health, Purulia, West Bengal 723101, India. ^11^Department of Microbiology, University of Calcutta, Kolkata, West Bengal 700019, India. ^12^Advanced Functional Nanomaterials, Energy and Environment Unit, Institute of Nano Science and Technology (INST), Phase X, SAS Nagar, Mohali, Punjab 160062, India. ^13^Department of Microbiology, Santiniketan Medical College, Gobindapur, Muluk, Bolpur, Birbhum, West Bengal 731204, India. ^14^Department of Economics, Raiganj University, North Dinajpur, West Bengal 733134, India. ^15^Department of Medical Microbiology, School of Medicine, Acibadem Mehmet Ali Aydınlar University, Ataşehir, Istanbul 34752, Turkey. ^16^Department of Otorhinolaryngology, Institute of Post Graduate Medical Education & Research (S.S.K.M. Hospital), Kolkata, West Bengal 700020, India. ^17^Centre for Nanotechnology Sciences, Raiganj University, North Dinajpur, West Bengal 733134, India.

**Correspondence:** amitent2004@gmail.com; amitmandal08@ gmail.com; [ikbal.agah.ince@gmail.com](mailto:ikbal.agah.ince@gmail.com)

| **Supplementary Table 1:** Correlations among Spatiotemporal variables. | | | | | |
| --- | --- | --- | --- | --- | --- |
|  | | Elevation | Temperature | Rainfall | Humidity |
| Elevation | Pearson Correlation | 1 | -.786^**^ | .724^**^ | -.808^**^ |
|  | Sig. (2-tailed) |  | .000 | .000 | .000 |
|  | N | 20 | 20 | 20 | 20 |
| Temperature | Pearson Correlation | -.786^**^ | 1 | -.531^*^ | .745^**^ |
|  | Sig. (2-tailed) | .000 |  | .016 | .000 |
|  | N | 20 | 20 | 20 | 20 |
| Rainfall | Pearson Correlation | .724^**^ | -.531^*^ | 1 | -.452^*^ |
|  | Sig. (2-tailed) | .000 | .016 |  | .046 |
|  | N | 20 | 20 | 20 | 20 |
| Humidity | Pearson Correlation | -.808^**^ | .745^**^ | -.452^*^ | 1 |
|  | Sig. (2-tailed) | .000 | .000 | .046 |  |
|  | N | 20 | 20 | 20 | 20 |
| N.B. **. Correlation is significant at the 0.01 level (2-tailed). | | | | | |
| *. Correlation is significant at the 0.05 level (2-tailed). | | | | | |
|  | | | | | |

(Source: Prepared by the authors, 2022 using SPSS v.24)


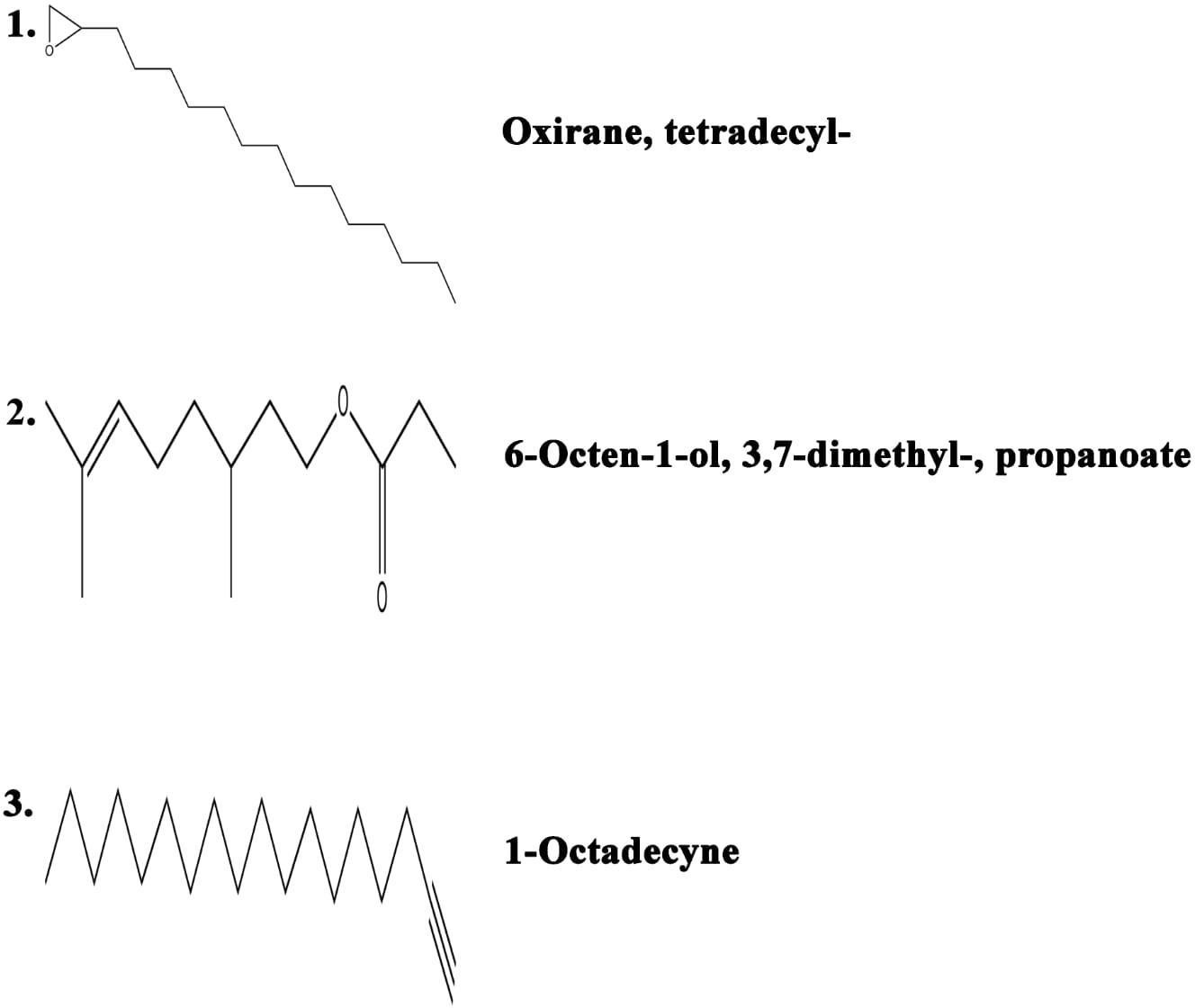


**Supplementary Figure 1:** Structure of Oxirane, tetradecyl-SS Hexadecane, 1,2-epox; 6-Octen-1-ol, 3,7-dimethyl-, propanoate; and 1-Octadecyne


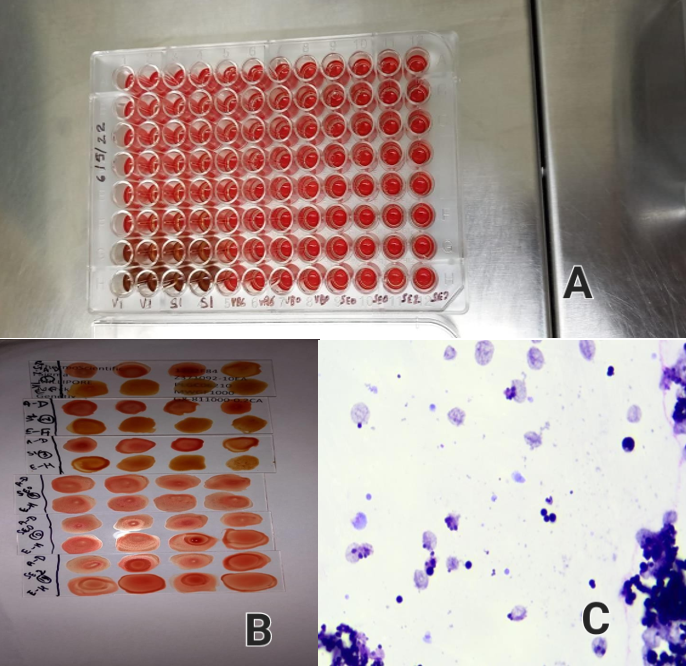


**Supplementary Figure 2:** In vitro antimalarial study: (A Microtiter plates with 96-wells exhibiting in vitro antiplasmodial activity of the methanolic leaf extract of *M. alba* S1, B. Preparation of blood slides, C. Microscopic field of CQS (3D7) strain of *P. falciparum* after treating with methanolic leaf extract).
